# Isolation and Identification of Sandfly-Borne Viruses from Sandflies Collected from June to August, 2019, in Yangquan County, China

**DOI:** 10.3390/v14122692

**Published:** 2022-11-30

**Authors:** Qinyan Wang, Qikai Yin, Shihong Fu, Jingxia Cheng, Xiuyan Xu, Jing Wang, Bin Wu, Xiaodong Tian, Yan Li, Jing Lu, Ying He, Fan Li, Kai Nie, Songtao Xu, Xiaoqing Lu, Huanyu Wang, Bin Wang, Guodong Liang

**Affiliations:** 1 Tongzhou Center for Disease Control and Prevention, Beijing 101199, China; 2 State Key Laboratory of Infectious Disease Prevention and Control, National Institute for Viral Disease Control and Prevention, Chinese Center for Disease Control and Prevention, Beijing 102206, China; 3 School of Public Health, Qingdao University, Qingdao 266071, China; 4 Shanxi Province Center for Disease Control and Prevention, Taiyuan 030012, China; 5 Yangquan Center for Disease Control and Prevention, Yangquan 045099, China

**Keywords:** sandfly, Wuxiang virus, Phlebovirus, spatial and temporal distribution

## Abstract

In Yangquan County, the sandfly-transmitted virus (Wuxiang virus) was first isolated from sandflies in 2018. However, relationships between the abundance and seasonal fluctuations of local sandflies and sandfly-transmitted viruses are unknown. Herein, we report that sandfly specimens were collected in three villages in Yangquan County, from June to August, 2019. A total of 8363 sandflies were collected (June, 7927; July, 428; August, 8). Eighteen virus strains (June, 18; July, 0; August, 0) were isolated in pools of Phlebotomus chinensis. The genome sequence of the newly isolated virus strain was highly similar to that of the Wuxiang virus (WUXV), isolated from sandflies in Yangquan County in 2018. Our results suggested that the sandfly-transmitted viruses, and the local sandfly population, are stable in Yangquan County, and that June is the peak period for the virus carried by sandflies in this area.

## 1. Introduction

The Phlebovirus genus belongs to the family Phenuiviridae and the order Bunyavirales [1]. In March 2020, the International Committee on Taxonomy of Viruses (ICTV) updated its classification list of sandfly viruses. Sixty Phlebovirus species were recognized, including the sandfly-transmitted Toscana and Toros viruses and the Rift Valley fever virus transmitted by mosquitoes. Furthermore, ‘sandfly fever Naples phlebovirus’ was renamed ‘Naples phlebovirus’, and the tick-borne ‘severe fever with thrombocytopenia syndrome virus’ and the ‘Heartland virus’, were reclassified from the Phlebovirus genus to the new genus Bandavirus [2,3].

The transmission vectors of phleboviruses include sandflies, mosquitoes, ticks, and other arthropods [4]. Phleboviruses transmitted by sandflies, such as sandfly fever Sicilian virus and Naples phlebovirus, can cause febrile disease, also referred to as “three-day fever” [5]. Viruses transmitted by sandflies have a significant impact on public health. Mediterranean coastal countries, such as Italy [6], Turkey [7], and Cyprus [8], are the main endemic areas for sandfly-transmitted viruses. These viruses are distributed mainly in tropical and semi-arid temperate regions, including the Mediterranean, North Africa, and Central and Western Asia [2,3]. Of phleboviruses, 37 species were first isolated in the Americas (2 in North America, 13 in Central America, 22 in South America), 11 in Africa (1 in West Africa, 2 in East Africa, 3 in Central Africa, 5 in North Africa), 8 in Asia (1 in East Asia, 7 in West Asia), and 4 in Europe (2 in Western Europe, 2 in Southern Europe). Furthermore, 37 phleboviruses are transmitted via sandflies, 5 via mosquitoes, 1 via ticks, and 17 via unknown viral transmission vectors [1].

In the summer of 2018, we isolated Wuxiang virus (WUXV) from sandfly specimens collected in Yangquan County, Shanxi Province, Central China. This was the first time the phlebovirus was isolated from sandfly specimens collected in rural areas of Yangquan County [9]. However, it is unclear whether WUXV was present in rural Yangquan County for a long time, or whether it only appeared in the local sandfly population occasionally. Moreover, the relationship between the population abundance of sandflies in local natural areas and the spatial and temporal distributions of WUXV carried by sandflies is unknown. To investigate the association between local sandfly populations and sandfly-borne viruses, we continuously collected sandfly specimens from six fixed specimen collection points in three villages in Yangquan County, from June to August 2019, to clarify the relationship between local sandflies and viruses and to further our understanding of the public health hazards associated with sandflies and the viruses they carry.

## 2. Materials and Methods

### 2.1. Samples

**Figure 1:**
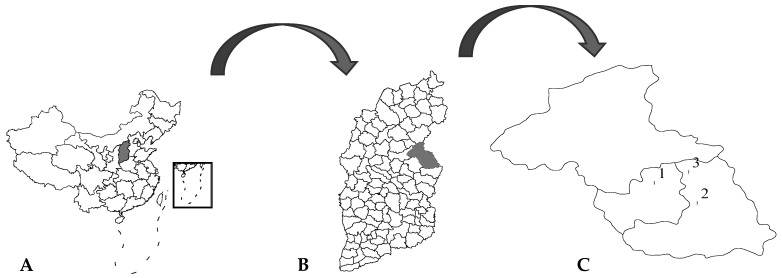
Specimen collection villages in Yangquan County, Shanxi Province, China in 2019. Note: (**A**) The location of Shanxi Province in China. (**B**) The location of Yangquan County in Shanxi Province. (**C**) The geographic location of the three collection villages in Yangquan County. The first sampling village (113°33′51″ east longitude, 37°59′48″ north latitude) is 18 km from Yangquan County municipality. The second sampling village (113°44′0″ east longitude, 37°54′24″ north latitude) is 21.3 km from Yangquan County municipality. The third sampling village (113°42′1″ east longitude, 38°2′41″ north latitude) is 28.8 km from Yangquan County municipality. The distances among the three sampled villages are 15–30 km.

**Figure 2:**
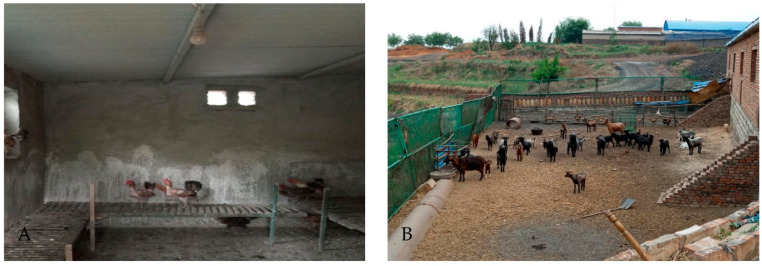
Photographs of representative insect breeding grounds and collected sandflies. Note: (**A**) Chicken pen. (**B**) Sheep pen.

Three villages in Yangquan county, Shanxi Province, China were selected for sampling (Figure 1) for the period June to August, 2019, and two fixed specimen collection points (chicken coops or sheep pens, shown in Figure 2A,B) per sampled village were selected, totaling six fixed sandfly specimen collection points. Each collection point was fitted with a blood-sucking insect collection tool (battery 12 V and 15 Ah, bulb 0.1 A, fan 0.14 A; MM200BL, Guangzhou Changsheng Chemical Technology Service Co., Ltd. Guangzhou, China. https://guangzhou.11467.com/info/8049209.htm (accessed on 27 November 2022)). The specimen collection period was from 18:00 of one day to 06:00 of the next day. All collected sandfly specimens were placed in a low-temperature refrigerator for 30 min and then transferred to an ice bath, where the sandflies were classified according to insect morphology, collection time, and collection environment, among other criteria. All collected specimens were stored in liquid nitrogen until laboratory testing [10,11,12,13].

### 2.2. Cells

BHK-21 cells (Golden hamster kidney cells) and C6/36 cells (Aedes albopictus oocytes) were cultured for viral infection analysis. BHK-21 cells were cultured in 90% Eagle’s minimal essential medium (laboratory preparation), supplemented with 7% fetal bovine serum (FBS; Invitrogen, Waltham, MA, USA), 1% penicillin and streptomycin (100 U/mL), 1% glutamine (30 g/L), and 1% NaHCO_3_, in a 37 °C incubator with 5% CO_2_. C6/36 cells were cultured in 89% Roswell Park Memorial Institute Medium 1640 (Invitrogen), supplemented with 10% FBS and 1% penicillin and streptomycin (100 U/mL), in a 28 °C incubator [10,11,12].

### 2.3. Virus Isolation

Pools of 50–100 sandflies were added to a glass grinder and washed twice with grinding fluid (93% Eagle’s minimal essential medium supplemented with 5% penicillin and streptomycin [100 U/mL], 1% glutamine [30 g/L], and 1% NaHCO_3_). Next, 1.5 mL grinding fluid was added to the samples, which were ground under ice bath conditions until the specimens were completely homogenized. The samples were centrifuged (4 °C, 12,000 rpm, 30 min), and 100 µL of the ground supernatant was inoculated with an 80% confluent monolayer of BHK-21 cells or C6/36 cells on culture plates (24-well plates, Corning Inc., New York, NY, USA). The inoculated BHK-21 and C6/36 cells were grown continuously and monitored for cytopathic effects (CPEs) under a microscope every 12 h. The supernatants of cell cultures exhibiting CPEs were collected and stored at −80 °C for viral identification. The supernatants of first-generation cells without CPEs were blindly passed through both BHK-21 and C6/36 cells for three generations and those that did not cause CPEs were discarded [11,12].

### 2.4. Viral RNA Extraction and cDNA Library Preparation

Total RNA was extracted from the grinding fluid of sandfly specimens and the supernatants of infected BHK-21 cells and C6/36 cells using the Viral RNA Mini Kit (QIAamp; Qiagen, Valencia, CA, USA), according to the manufacturer’s instructions. The extracted RNA was immediately incubated in a 65 °C water bath for 10 min and then quickly transferred to an ice bath for 2 min. RNA (32 µL) was added to the first-strand reaction tube of the Ready-To-Go kit (GE Healthcare, Little Chalfont, Buckinghamshire, UK). The reaction tube was incubated at room temperature for 1 min, and then 1 µL random primer (pd(N)6) (TaKaRa, Shiga, Japan) was added, before further incubation at 37 °C for 1 h. The total volume of the resulting cDNA library was 33 µL, which was used immediately or stored at −40 °C for further use [10,14,15].

### 2.5. Preliminary Identification of Virus Isolates

WUXV-specific primers (S1-1F/S1-458R; Supplementary Table S1) were used to screen the virus isolates. The viral genes were amplified by polymerase chain reaction (PCR) in a 25 µL reaction volume comprising cDNA template, 2× GoTaq^®^ GreenMasterMix (Promega, Madison, WI, USA), and 10 µmol/L each primer (forward and reverse). After PCR, 5 µL of the amplification product was examined by 1% agarose gel electrophoresis. The nucleotide sequences of the WUXV-positive amplification products were then determined by first generation Sanger sequencing [10,14,15].

### 2.6. Viral Gene Amplification and Sequencing

Viral genes were amplified by first generation Sanger sequencing by PCR in a 25 µL reaction volume comprising cDNA template, 2× GoTaq^®^ Green Master Mix, and 10 µmol/L each of the forward and reverse primers. The primers used were specific to the Wuxiang virus. The PCR products (5 µL) were analyzed by 1% agarose gel electrophoresis. The nucleotide sequences of the virus-positive amplification products were determined [10,14,15]. The primers used to amplify the large (L), medium (M), and small (S) segments of the WUXV genome are listed in Supplementary Table S1.

### 2.7. Nucleotide Sequence Analysis

The viral nucleotide sequences were aligned using the basic local alignment search tool (BLAST; National Center for Biotechnology Information [NCBI]). Seqman software (DNAStar, Madison, WI, USA) was used to assemble the nucleotide sequences and analyze read quality. Nucleotide multiple sequence alignment was performed using BioEdit software (version 7.0, Tom Hall, CA, USA). MEGA6.0 software was used to conduct phylogenetic analyses using the neighbor-joining method with a bootstrap value of 1000, and identity analyses of nucleotide and amino acid sequences were performed using MegAlign software [10,11,15]. Except for the 18 viruses isolated in this study, all viral gene sequences were obtained from the GenBank database (NCBI). The name, GenBank accession number, time of isolation, country (or region) of isolation, and transmission vector of each virus are shown in Supplementary Table S2.

### 2.8. Genetic Identification of Sandfly Species

The DNA of WUXV-positive sandfly specimens was extracted and subjected to PCR amplification targeting the mitochondrial cytochrome c oxidase I gene [11] using the forward primer LCO1490 (5′-GGTCAACAAATCATAAAGATATTGG-3′) and reverse primer HC02198 (5′-TAAACTTCAGGGTGACCAAAAAATCA-3′). The nucleotide sequences of the PCR products were determined and then subjected to a BLAST search against the GenBank database to determine the sandfly species [10,16].

### 2.9. Minimum Infection Rate

Assuming that each positive pool contained only one infected sandfly, the minimum infection rate was calculated as follows: minimum infection rate of 1000 sandflies = number of positive specimen pools (number of infected sandflies)/total treated sandflies × 1000 [17].

## 3. Results

### 3.1. Sandfly Specimen Collection

We conducted four specimen collections at six fixed collection points in three villages in Yangquan County, Shanxi Province, China (shown in Figure 1) on 11 June 2019, 25 June 2019, 20 July 2019, and 17 August 2019. A total of 8363 sandflies were obtained from the four collections (Table 1).

### 3.2. Virus Isolation and Preliminary Identification

**Figure 3:**
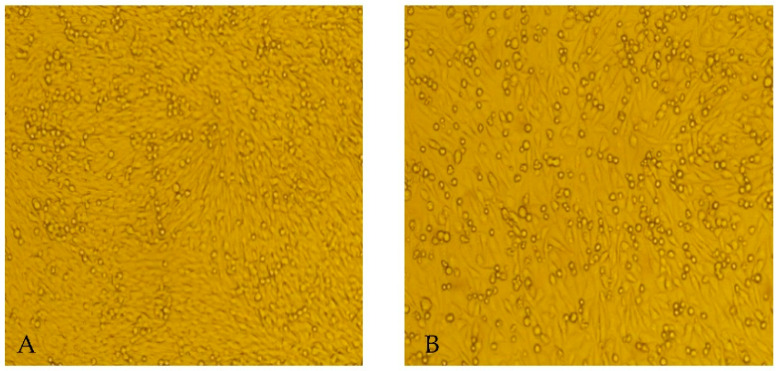
Cytopathic effects (CPEs) of the SXYQ1944-2 virus isolate on BHK-21 cells. Note: (**A**) BHK-21 cells cultured for 3 days under standard conditions. (**B**) BHK-21 cells 3 days after inoculation with ground sandfly supernatant containing the SXYQ1944-2 virus isolate. Compared with the BHK-21 cultures in (**A**), those in (**B**) had fewer adherent cells and more shrunken and exfoliated cells. 200× magnification.

The 8363 collected sandfly specimens were divided into 106 pools for grinding treatment. Of the 106 sandfly supernatant pools, 18 triggered CPEs in BHK-21 cells and were stably passaged (Table 2; Figure 3). BHK-21 cells inoculated with the virus isolate SXYQ1944-2 exhibited CPEs, such as cell shrinkage and shedding, on day 3 after inoculation (Figure 3). Moreover, no CPEs were observed in C6/36 cells inoculated with ground sandfly. All 18 samples with CPEs tested positive using PCR.

### 3.3. Sandfly Species Identification

To identify the sandfly species in which WUXV was detected, the 18 WUXV-positive sandfly pools were subjected to genetic testing. We found that 18 WUXV-positive sandfly species were *Phlebotomus chinensis*. No sequence information for other sandfly species was found in 18 pools.

### 3.4. Molecular Characteristics of the Viruses

#### 3.4.1. Nucleotide Sequence and Homology of Viral Genes

We determined the nucleotide sequences of the coding regions of the L, M, and S segments of the isolated virus strains. The number of nucleotides (number of amino acids) of the coding regions of the L, M, NS, and N genes of the 18 virus isolates were 6273 nt (2090 aa), 4089 nt (1362 aa), 783 nt (260 aa), and 741 nt (246 aa), respectively. The L, M, and S segment lengths were the same between the WUXV strain isolated in the present study (SXYQ1944-2) and that isolated previously (SXWX1813-2) (Table 3).

Nucleotide sequence alignment of the M, NS and N segments of each of the 18 virus isolates revealed nucleotide and amino acid sequence identities of 96.5–99.8% (97.1–99.8%), 94.6–100% (97.3–99.99%) and 98.1–100% (98.8–99.6%) among the isolates, respectively (Supplementary Tables S3–S5). Homology analyses, comparing the novel WUXV strain SXYQ1944-2 with other sandfly viruses, are shown in Table 3. The nucleotide and amino acid sequences of the L and M segments of SXYQ1944-2 and SXWX1813-2 shared 97.6% (99.2%) and 96.7% (97.7%) homology, respectively. A comparison of SXYQ1944-2 with Toros virus isolated from sandflies in Turkey revealed 76.9% (88.0%) and 72.0% (75.3%) nucleotide (amino acid) homologies of the L and M segments, respectively. These results suggested that SXYQ1944-2 isolated from sandflies in Yangquan County was the same viral species as SXWX1813-2 isolated from another area in China.

#### 3.4.2. Phylogenetic Analysis of the Isolated Virus

**Figure 4:**
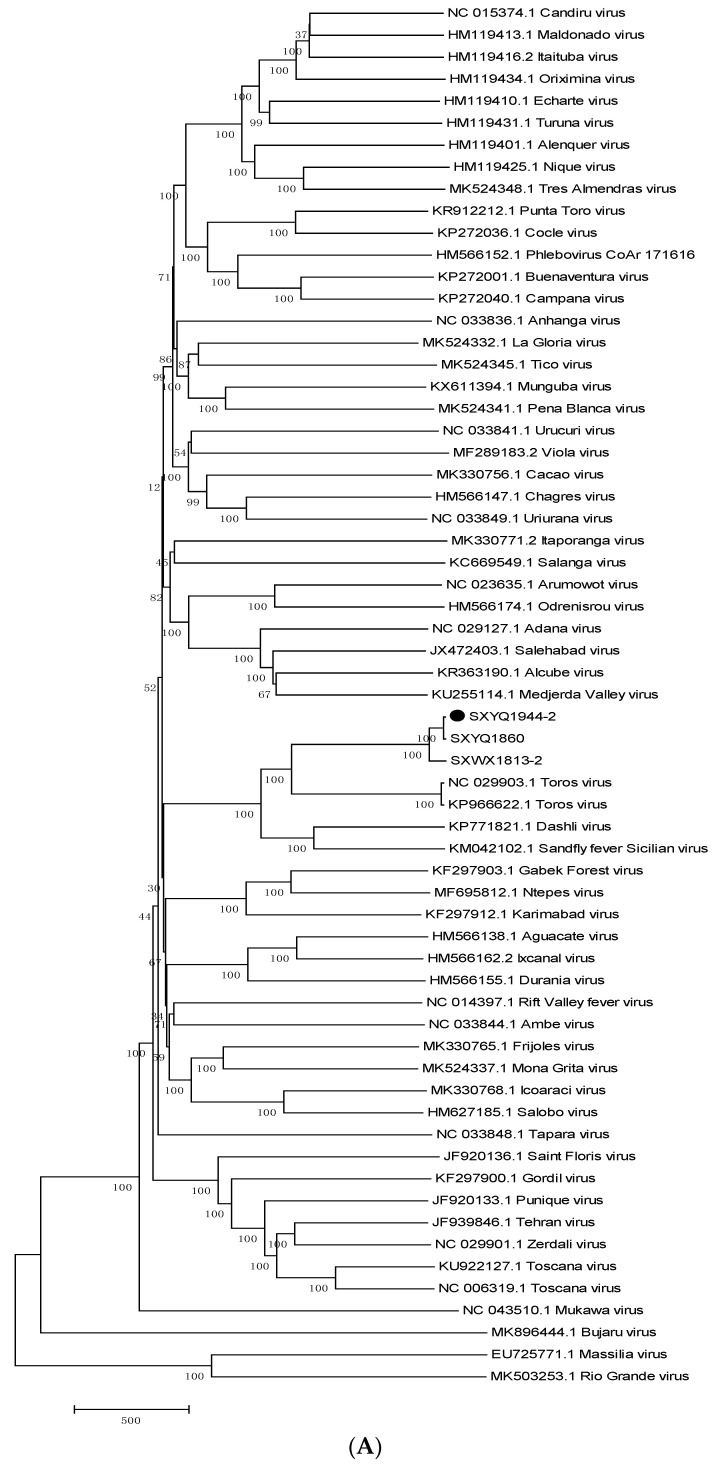

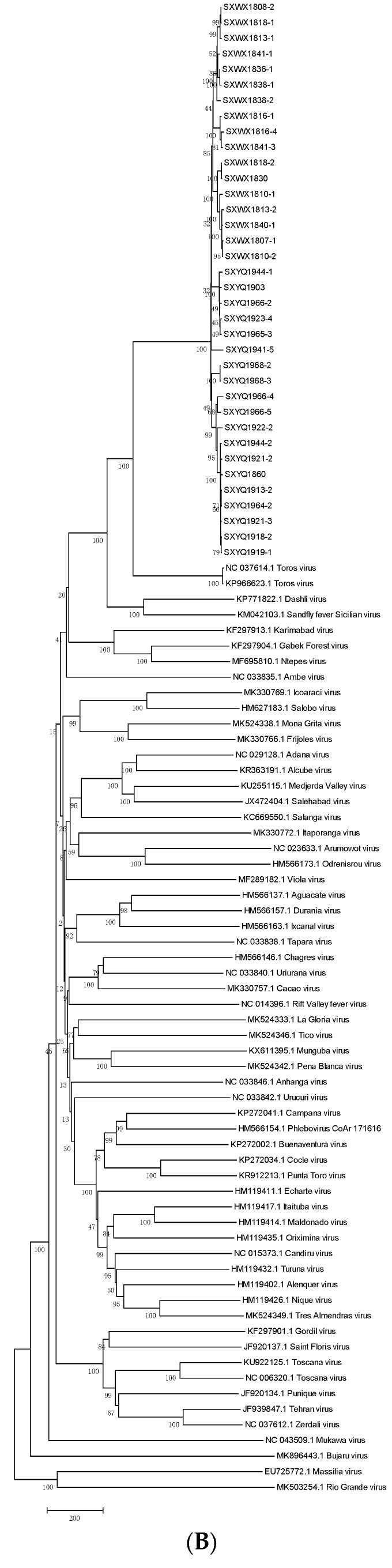

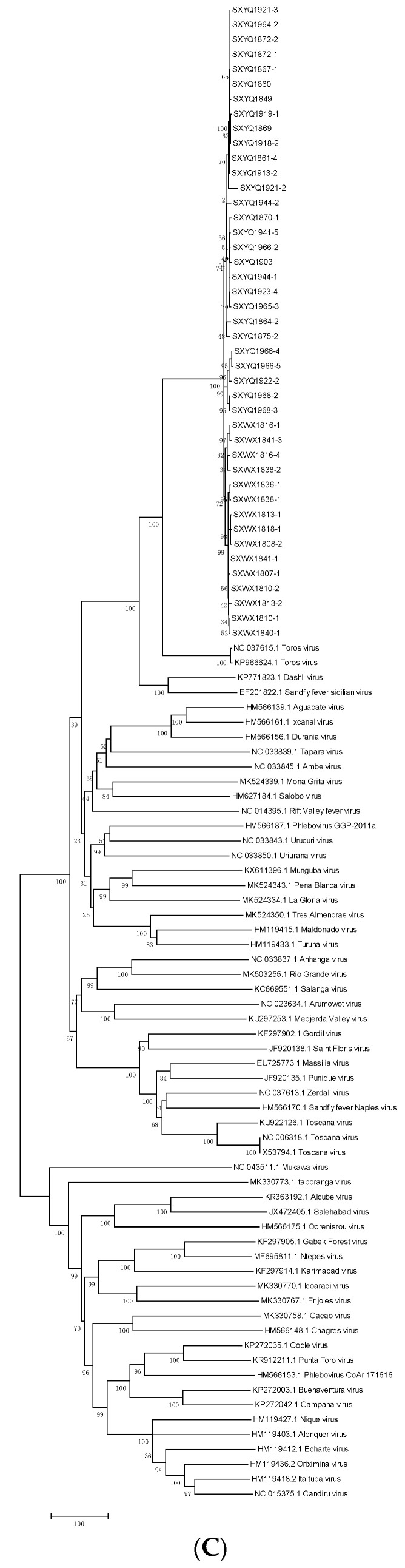
Molecular phylogenetic analysis of viral gene segments. Note: (1) A comparison of the gene sequences and related information of phleboviruses recently identified by the International Committee on Taxonomy of Viruses in 2020 via molecular genetic phylogenetic analysis. (2) (**A**) Phylogenetic analysis of the viral L segment sequence. (**B**) Phylogenetic analysis of the viral M segment sequence. (**C**) Phylogenetic analysis of the viral S segment sequence. MEGA6.0 software was used to perform the phylogenetic analysis, using the neighbor-joining method with a bootstrap value of 1000.

The genome sequences of the 18 virus strains isolated in this study were subjected to molecular phylogenetic analysis by comparing the L, M, and S genomic segments with those of 60 sandfly viruses recognized by ICTV. The 18 viruses isolated in this study clustered in the same phylogenetic branch as the WUXV strain SXWX1813-2 (Figure 4), providing further evidence that the novel isolate was closely related to WUXV.

## 4. Discussion

In this study, a group of WUXV virus strains was isolated from sandfly specimens collected in rural areas of Yangquan County in 2019.

### 4.1. Ecological Characteristics of Sandflies in Yangquan County

Yangquan County (112°5′–114°4′ east longitude, 37°40′–38°31′ north latitude) is located in Shanxi Province in Central China on the west side of the central Taihang Mountains, with an average elevation of 700–1700 m and a warm temperate continental climate. Ecological environments that are arid, with low rainfall and high altitudes, are suitable for the growth and reproduction of sandflies, mosquitoes, and other blood-sucking insects [10,18]. However, nighttime temperatures in April and October are around 0 °C, which is not conducive to sandfly growth. Our research team collected only two sandflies in Yangquan County in early May 2019; we did not include these data in Table 1 because of the low number of sandflies collected. May is likely the first month of the season for sandfly populations in rural Yangquan County. The number of local sandflies collected in this study peaked in June, with the highest number of sandflies collected on 11 June 2019 (1420 per collector per night), after which the abundance of sandflies gradually decreased in July, reaching the lowest value (two sandflies) in August. *Phlebotomus chinensis* is the dominant species of sandflies in Yangquan County [19,20,21]. This was supported by the molecular species identification analysis performed in this study, which revealed that the collected sandflies were *P. chinensis*. *P. chinensis* sandflies reside in near-wild habitats, breed in the wild, and inhabit nearby village livestock houses, where they feed on the blood of cows, donkeys (mules), chickens, and domestic dogs [22]. Pigs are the main source of blood for *P. chinensis* in Sichuan Province, Western China [23]. Recently, the blood obtained by *P. chinensis* in Yangquan County, Shanxi Province, China was examined, revealing that 88.9% and 66.7% of sandflies contained chicken and human blood, respectively [24], suggesting that chickens and humans are the main blood sources for local *P. chinensis* in Yangquan County. Given that multiple virus strains were isolated from sandflies in Yangquan County, a seroepidemiological investigation into sandfly-transmitted viruses in local human and animal populations is needed. This would enable evaluation of the infection prevalence and public health significance of sandfly-transmitted viruses.

### 4.2. Molecular Biological Characteristics of Viruses Transmitted by Sandflies in Yangquan County

We performed molecular phylogenetic analyses of the 60 Phlebovirus species recognized by ICTV in 2020 and the 18 virus strains isolated from sandflies in Yangquan County in this study. We found that the L, M, and S genomic segments each formed a common phylogenetic branch with a WUXV strain, previously isolated in China [10], and a virus isolated from sandflies in Yangquan County in 2018 [9] (Figure 4A–C), suggesting that the viruses isolated from sandflies in Yangquan County in the present study were WUXV. In addition, our molecular phylogenetic analysis results suggested that the WUXV, isolated from the *P. chinensis* specimens in this study, was closely related to the Toros virus isolated from sandflies in Turkey in 2012 [25].

The PCR primers used to amplify the original WUXV genome were initially applied to amplify the genome of the virus strain isolated in this study. The nucleotide sequences of the three genomic segments of sandfly virus isolated here and the original WUXV isolated in the Wuxiang area exhibited differences, which might explain the poor amplification of the strains of newly isolated WUXV genomes using the primers designed for the original WUXV isolate (Supplementary Table S1). Moreover, the phylogenetic analysis revealed differences in the branch lengths between the virus isolates (Figure 4B,C), which suggested that the WUXV genomes were constantly evolving.

## 5. Conclusions

In this study, we combined field surveys of rural sandflies in Yangquan county with cell culture and molecular methods to isolate and identify sandfly viruses. The primary host species of the WUXV strain, and the prevalence of the virus in humans and animals, requires further study. The bloodsucking habits of sandflies also require further exploration. *Leishmania* spp., a parasite that causes the severe sandfly-transmitted disease kala-azar, is endemic in Yangquan County [26]. The potential existence of Leishmania and WUXV co-infections in rural areas merits further investigation.

## Figures and Tables

**Table 1:** Specimen collection in Yangquan County, Shanxi Province in 2019.

Date	1				2				3				Total (Sandfly Abundance)
	Chicken Pen	Sheep Pen	Mule Pen	Subtotal (Sandfly Abundance)	Chicken Pen	Sheep Pen	Mule Pen	Subtotal (Sandfly Abundance)	Chicken Pen	Sheep Pen	Mule Pen	Subtotal (Sandfly Abundance)	
6.11	2289	1005	/	3294 (1647)	/	371	1222	1593( 796.5)	572	221	/	793 (396.5)	5680 (1420)
6.25	387	397	/	784 (392)	/	807	85	892 (446)	110	461	/	571 (285.5)	2247 (561.75)
7.20	102	27	/	129 (64.5)	/	24	31	55 (27.5)	122	122	/	244 (122)	428 (107)
8.17	4	2	/	6 (3)	/	0	2	2 (1)	0	0	/	0 (0)	8 (2)
Total (Sandfly abundance)	2782 (695.5)	1431 (357.75)	/	4213 (526.63)	/	1202 (300.5)	1340 (335)	2542 (317.75)	804 (201)	804 (201)	/	1608 (201)	8363 (522.69)

Note: 1, 2 and 3 represent each of the three sampling villages. Each sampling point (sheep pen, chicken pen, etc.) had one specimen collector. “/” indicates that the collection village lacked a mule or chicken pen. Sandfly abundance is the number of sandflies captured by each blood-sucking insect collector per night.

**Table 2:** Sandfly viruses isolated in this study (Yangquan County, 2019).

Strain Number	Date	Collection Site	Breeding Places	Number of *Phlebotomus chinensis*	CPE/Gene Amplification							
					BHK-21 Cell	WUXV			C6/36 Cell	WUXV		
						L	M	S		L	M	S
SXYQ1921-2	11 June 2019	1	Chicken pen	85	+	-	MW805215	MW805196	-	-	-	-
SXYQ1921-3				85	+	-	MW805216	MW805198	-	-	-	-
SXYQ1919-1				73	+	-	MW805214	MW805197	-	-	-	-
SXYQ1922-2				100	+	-	MW805217	MW805199	-	-	-	-
SXYQ1923-4				100	+	-	MW805218	MW805200	-	-	-	-
SXYQ1903			Sheep pen	100	+	-	MW805211	MW805193	-	-	-	-
SXYQ1913-2				75	+	-	MW805212	MW805194	-	-	-	
SXYQ1918-2				100	+	-	MW805213	MW805195	-	-	-	-
SXYQ1941-5		2	Mule pen	91	+	-	MW805219	MW805201	-	-	-	-
SXYQ1944-1		3	Chicken pen	88	+	-	MW805220	MW805202	-	-	-	-
SXYQ1944-2				89	+	MW805191	MW805210	MW805192	-	-	-	-
SXYQ1964-2	25 June 2019	1	Sheep pen	81	+	-	MW805221	MW805203	-	-	-	-
SXYQ1965-3		2	Sheep pen	90	+	-	MW805222	MW805204	-	-	-	-
SXYQ1966-2				89	+	-	MW805223	MW805205	-	-	-	-
SXYQ1966-4				89	+	-	MW805224	MW805206	-	-		-
SXYQ1966-5				89	+	-	MW805225	MW805207	-	-	-	-
SXYQ1968-2		3	Sheep pen	77	+	-	MW805226	MW805208	-	-	-	-
SXYQ1968-3				77	+	-	MW805227	MW805209	-	-	-	-

Note: ‘+’ indicates the presence of CPEs. ‘-’ indicates the absence of CPEs. 1, 2, and 3 represent the three sampling villages.

**Table 3:** Viral genome sequence homology.

Virus Strains	L Segment		M Segment	
	RdRp		GP	
	nt (%)	aa (%)	nt (%)	aa (%)
SXYQ1944-2	6273	2090	4089	1362
SXWX1813-2	6273 (97.6)	2090 (99.2)	4089 (96.7)	1362 (97.7)
TORV (213/Turkey/2012)	6273 (76.9)	2090 (88.0)	4080 (72.0)	1359 (75.3)
TORV (292/Turkey/2012)	6273 (76.9)	2090 (88.0)	4081 (72.0)	1359 (75.2)
CFUV (Pa Ar 814/Greece/1981)	6273 (76.5)	2090 (88.1)	4080 (71.3)	1359 (75.9)
SFSV (Ethiopia-2011/Ethiopia/2011)	6273 (71.9)	2090 (78.4)	4026 (62.7)	1341 (57.1)
SFSV (U30500)	/	/	4026 (61.7)	1341 (56.5)
DASHV (131/Iran/2011)	6273(72.3)	2090 (79.2)	4029 (65.2)	1342 (58.6)
RVF (35/74/ South Africa/1974)	6279(59.5)	2092 (56.0)	3594 (33.4)	1197 (5.3)
SFNV(HM566170)	6288(59.4)	2095 (53.7)	3972 (32.4)	1323 (33.1)

Note: “/” indicates that the sequence is not available in the GenBank database.

## Data Availability

All available data are provided in the manuscript and Supplementary Materials.

## Supplementary Materials

The following are available online at https://www.mdpi.com/article/10.3390/v14122692/s1, Supplementary Table S1: Amplification primers for the WUXV genome, Supplementary Table S2: Information on the virus strains analyzed in this study, Supplementary Table S3: Nucleotide and amino acid homologies of the M segment of sandfly viruses isolated in Yangquan County, Supplementary Table S4: The homology analysis of nucleotide (amino acid) sequence of NS gene of WUXV viruses isolated in Yangquan County, Supplementary Table S5: The homology analysis of nucleotide (amino acid) sequence of N gene of WUXV viruses isolated in Yangquan County.
