# Supplementary material for: Isolation and Identification of Sandfly-Borne Viruses from Sandflies Collected from June to August, 2019, in Yangquan County, China
Source: Viruses. 2022 Nov 30;14(12):2692. doi: 10.3390/v14122692 (PMC9782482; doi:10.3390/v14122692)
Supplement: Supplementary file 1 [file viruses-14-02692-s001.zip › viruses-2051468-supplementary.pdf]

Supplement table S1. Amplification primers for the WUXV genome.

Primers	Amplified fragment	Sequence information 5'-3'	Amplified fragment(bp)
L1-9F	L	GCGCCTGTACATCATGAATTC	315
L1-323R		CTCCTTTTCCCGAAGACACTG	
★W2L2-172F	L	GAGCACTTTGACGTGGGCTC	833
★W2L2-1004R		GCAGGGACTTGTACTGTGCT	
★W2L3-857F	L	TCGCTGGAGCTATGGAGGAT	834
★W2L3-1690R		TTGAGCAGGTGGGCTTGATT	
★W2L4-1473F	L	CCACCAGCCCACTCTAATCAA	923
★W2L4-2395R		ACTCGTCACTTTCTGGCCTC	
★W1L5-2057F	L	TGTCACAGCCAGAAGTACCA	600
★W1L5-2656R		GCCTGTCCCTGGAATACGAC	
★W1L6-2369F	L	AGGACCAGAGGCCAGAAAGT	703
★W1L6-3071R		GGGTGCCATCTAGGATGTGT	
★W1L7-2855F	L	CCAGATCTGTGGGTCGGTTC	809
★W1L7-3633R		CACCTCAGGCAAACTGCAAC	
★W1L8-3259F	L	ACCACTGGGATGATGCAAGG	796
★W1L8-4054R		TCAGTACTATCGCCCCACCA	
★W1L9-3837F	L	GAGATGGAGGGATCCAGGAC	885
★W1L9-4721R		AACGGGCTCCTTTGAAGAGT	
★W1L10-4521F	L	AGAGGCCACACAGACTAGGA	625
★W1L10-5145R		AAACCCACCCACTATGCCAG	
★W1L11-5002F	L	TTCGACACTCCTGCAATAGACA	715
★W1L11-5716R		CCTTGCAGTAGACCCAGCAA	
★W1L12-5519F	L	AGCTCCTTGTCAACAGATCCA	857
★W1L12-6375R		ACATTTGTGCTGCAAAGGGG	
L9-6134F	L	GAGATGTCAGGAAACTTATTGG	265
L9-6398R		TAGCCCATGGGACTAAGCTATACA	
M1-9F	M	CTGGTGCATTAAATGTTTGA	185
M1-193R		GGCATCTTAGCCATCTCGTA	
★W1M2(1)-60F	M	TGACGTTCGTCTGGACTCATC	306
★W1M2(1)-366R		GAGTGCTCCAGCCATCATGT	
★W1M2(2)-217F	M	AAGTTCATGACAGCAGAGAACG	442
★W1M2(2)-659R		TGCTTTTCAACCTCCTGCTTCT	
M3-523F	M	GTAGCCGTCAGTTAGAAGAG	938
M3-1460R		CATCGGTCCATTATAGTCAG	
★W2M4-1246F	M	CCCAGAGGAGTGCCCTCAAT	525
★W2M4-1770R		TTCACTGTCGCAAGCGTACT	
★W1M5-1431F	M	TGGGCCGATGGAGATTCCTA	566
★W1M5-1996R		TGGCAGCTGATATCTTGAATCC	
M6-1865F	M	ACCCCAGTGTGTGGGATTTG	958
M6-2822R		CACTCCGTATTGGGTGGTCC	
★W2M7-2643F	M	GGTGTGGTCAACGTGAAAGC	994
★W2M7-3636R		TTCTAGCACCCTCGTTGCAG	
M8-3507F	M	ACAGTGCAAGCCCTAACCTC	574
M8-4080R		TGACCATCCCGCTGACTCTA	

M9-3920F	M	CAAGGCTGGAGACTGGAGTTTATGGA	403
M9-4322R		TTGAGTTGCGAATGACACAAAGACCG	
S1-1F	S	ACACAAAGTCCCCCTAGTAAAG	458
S1-458R		GAGACATCCTCTCTTGCATCTT	
★W3S2-356F	S	GGCCTTTTGGATTCCCTGACT	652
★W3S2-1007R		CTGCTGCTATCAACAGCTCCT	
S3-875F	S	AGCCCTCATCCCACCCCAATTTATGG	336
S3-1210R		CCTAGCTTTGCTGGTCTGATTGATCC	
★W1S4-916F	S	GCTAGCCCTAGCACTGGATG	517
★W1S4-1432R		GGCCTTGACTCGAGGAAACA	
S5-1333F	S	CTCTTGTCAGTGTTAGCCCGTTCC	199
S5-1531R		AACCTGATTAAGTTAGGCGGAAGT	
S6-1441F	S	TCTTCTTTGGCTTGTTACCTC	247
S6-1687R		AGCTCCCTAGTACGTTCTTGA	

Note: L, M and S represent the L, M and S genes of WUXV, respectively; Initially, we used previously published primers [10] to amplify the L, M, and S genomic segments of the virus strains isolated in this study, however, seven of the nine primer pairs designed to amplify the L segment did not generate PCR products (expected fragment size: 5693nt). Similarly, four primer pairs targeting the M segment and two primer pairs targeting the S segment did not generate PCR products (expected fragment sizes: 2700 nt and 1079 nt, respectively). Therefore, we redesigned the primers that did not produce amplification products, the redesigned primers are labelled with “★”.

Supplement Table S2 Information on the virus strains analyzed in this study.

No.	Virus	Strain	Year	Country	Host	Genbank number		
						L	M	S
1	Bujaru virus	USA 847-32	1954	USA	Culex tarsalis	MK896444	MK896443	MK896442
2	Candiru virus	/	1967	Brazil	Homo sapiens	NC_015374	NC_015373	NC_015375
3	Frijoles virus	VP-161A	1969	Panama	Lutzomyia sp	MK330765	MK330766	MK330767
4	Mukawa virus	MKW73	2013	Japan	Ixodes persulcatus	NC_043510	NC_043509	NC_043511
5	Punta Toro virus	Balliet	1966	Panama	Homo sapiens	KR912212	KR912213	KR912211
6	Rift Valley fever virus	ZH-548	1977	Egypt	human sera	NC_014397	NC_014396	NC_014395
7	Salehabad virus	I-81	1959	Iran	Phlebotomus sp.	JX472403	JX472404	JX472405
8	Naples phlebovirus	Toscana ISS.Phl.3	1944	Italy	sandfly	NC_006319	NC_006320	NC_006318
9	Adana virus	195	2012	Turkey	Phlebotomus sp.	NC_029127	NC_029128	NC_029129
10	Aguacate virus	VP-175A	1969	Panama	Lutzomyia sp.	HM566138.1	HM566137	HM566139.1
11	Alcube virus	S20	2007	Portugal	Phlebotomus perniciosus	KR363190	KR363191.1	KR363192.1
12	Alenquer virus	/	1976	Brazil	Homo sapiens	HM119401	HM119402	HM119403
13	Ambe virus	BeAr407981	1982	Brazil	Phlebotominae sp.	NC_033844	NC_033835	NC_033845
14	Anhanga virus	BeAn46852	1962	Brazil	Choloepus didactylus	NC_033836	NC_033846	NC_033837
15	Arumowot virus	/	1963	Sudan	Mosquito	NC_023635	NC_023633	NC_023634
16	Buenaventura virus	CoAr3319	1964	Colombia	sandfly	KP272001	KP272002	KP272003
17	Cacao virus	VP-437R	1970	Panama	Nyssomyia trapidoi	MK330756	MK330757	MK330758
18	Campana virus	VP-334K	1970	Panama	sandfly	KP272040	KP272041	KP272042
19	Chagres virus	/	/	Panama	/	HM566147	HM566146	HM566148
20	Cocle virus	GML244915	2009	Panama	Homo sapiens	KP272036	KP272034	KP272035
21	Dashli	131	2011	Iran	sand fly	KP771821	KP771822	KP771823
22	Durania virus	Co Ar 171162	1986	Colombia	Lutzomyia sp.	HM566155	HM566157	HM566156

23	Echarate virus	/	1998	Peru	Homo sapiens	HM119410	HM119411	HM119412
24	Gabek Forest virus	Sud AN 754-61	1961	Sudan	Acomys cahirinus	KF297903	KF297904	KF297905
25	Gordil virus	Dak ANBr 496d	1984	Central African Republic	Lemniscomys striatus	KF297900	KF297901	KF297902
26	Icoaraci virus	BeAn24262	1960	Brazil	rodent	MK330768	MK330769	MK330770
27	Itaituba virus		1971	Brazil	opossum	HM119416	HM119417	HM119418
28	Itaporanga virus	original	1962	Brazil:	sentinel mouse	MK330771	MK330772	MK330773
29	Ixcanal virus	CA Ar 170897	1982	Guatemala	Lutzomyia sp.	HM566162	HM566163	HM566161
30	Karimabad virus	I-58	1959	Iran	Phlebotomus sp.	KF297912	KF297913	KF297914
31	La Gloria virus	SP0584-PA-2014	2014	Panama	Psychodopygus panamensis	MK524332	MK524333	MK524334
32	Lara phlebovirus	Phlebovirus GGP-2011a	/	Venezuela	sentinel hamster	/	/	HM566187
33	Leticia virus	PhlebovirusCoAr171616	1987	Colombia	Sandfly	HM566152	HM566154	HM566153
34	Maldonado virus	FMD 0077	2004	Peru	Homo sapiens	HM119413	HM119414	HM119415
35	Massilia virus	W	2005	France	Phlebotomus perniciosus	EU725771	EU725772	EU725773
36	Medjerda Valley virus	T131	2010	Tunisia	Phlebotomus (Larroussius) sp.	KU255114	KU255115	KU297253
37	Mona Grita virus	SP0260-PA-2014	2014	Panama	Nyssomyia trapidoi	MK524337	MK524338	MK524339
38	Munguba virus	BeAr389707	1980	Brazil	Nyssomyia umbratilis	KX611394	KX611395	KX611396
39	Nique virus	/	1972	Panama	sandfly	HM119425	HM119426	HM119427
40	Ntepes virus	MRG54-KE-2014	2014	Kenya	Sergentomyia sp.	MF695812	MF695810	MF695811
41	Odrenisrou virus	/	1980	Côte d'Ivoire	Mosquito	HM566174	HM566173	HM566175
42	Oriximina virus	/	1980	Brazil	Sandfly	HM119434	HM119435	HM119436
43	Pena Blanca virus	SP1683-PA-2014	2014	Panama	Psychodopygus panamensis	MK524341	MK524342	MK524343
44	Punique virus	P1_B4_2008	2008	Tunisia	Phlebotomus perniciosus	JF920133	JF920134	JF920135
45	Rio Grande virus	TBM3-204	1973	USA	Neotoma micropus	MK503253	MK503254	MK503255
46	Saint Floris virus	Dak ANB 512	1971	Central African Republic	Tatera sp	JF920136	JF920137	JF920138
47	Salanga virus	AnB 904a	1971	Central African Republic	Aethomys medicatus	KC669549	KC669550	KC669551
48	Salobo virus	/	1997	Brazil	/	HM627185	HM627183	HM627184

49	Sandfly Sicilian virus	Ethiopia 2011	2011	Ethiopia	Human	KM042102	KM042103	KM042104（N）<br>KM042105（NS）
		Sabin	1943	Italy	Human	/	/	EF201822
50	Tapara virus	BeAr413570	1983	Brazil	Phlebotominae sp.	NC_033848	NC_033838	NC_033839
51	Tehran virus	I-47	1976	Iran	Phlebotomus perfiliewi	JF939846	JF939847	JF939848
52	Tico virus	SP0157-PA-2013	2013	Panama	Lutzomyia sp.	MK524345	MK524346	MK524347
53	Toros virus	213	2012-2013	Turkey	Phlebotomus sp.	NC_029903	NC_037614	NC_037615
		292	2012-2013	Turkey	Phlebotomus sp.	KP966622	KP966623	KP966624
54	Toscana virus	H4906	2004	France	Homo sapiens	KU922127	KU922125	KU922126
55	Tres Almendras virus	SP0412-PA-2013	2013	Panama	Psychodopygus panamensis	MK524348	MK524349	MK524350
56	Turuna virus	/	1978	Brazil	sandfly	HM119431	HM119432	HM119433
57	Uriurana virus	BeAr479776	1985	Brazil	Phlebotominae sp.	NC_033849	NC_033840	NC_033850
58	Urucuri virus	BeAn100049	1966	Brazil	Proechimys guyannensis	NC_033841	NC_033842	NC_033843
59	Viola virus	BR/MT_PanAr2015	2015	Brazil	Lutzomyia sp.	MF289183	MF289182	/
60	Zerdali virus	37	2012-2013	Turkey	Phlebotomus sp.	NC_029901	NC_037612	NC_037613

Note: "/" represents no available sequence data in GenBank.

Supplement table S3. Nucleotide and amino acid homologies of the M segment of sandfly viruses isolated in Yangquan County.

NO	Strain	Strain																	
		1	2	3	4	5	6	7	8	9	10	11	12	13	14	15	16	17	18
1	SXYQ1944-2	***	96.9	99.7	99.7	99.7	99.7	99.7	98.8	96.9	96.7	96.8	99.8	96.9	96.9	98.4	98.6	97	97.1
2	SXYQ1903	98.3	***	96.9	96.8	96.8	96.9	96.8	96.6	99.3	96.5	99.2	96.9	99.6	99.4	97.1	96.9	96.8	96.7
3	SXYQ1913-2	99.6	98.2	***	99.7	99.6	99.7	99.6	98.7	96.9	96.6	96.7	99.8	96.9	97	98.4	98.5	97	97.1

4	SXYQ1918-2	99.6	98	99.6	***	99.8	99.7	99.7	98.8	96.8	96.6	96.8	99.8	96.9	96.9	98.5	98.6	97	97
5	SXYQ1919-1	99.6	97.9	99.4	99.6	***	99.6	99.7	98.7	96.9	96.6	96.7	99.7	96.9	96.9	98.4	98.5	97	97
6	SXYQ1921-2	99.6	98.1	99.7	99.4	99.3	***	99.6	98.7	96.9	96.6	96.7	99.8	96.9	96.9	98.4	98.5	97	97
7	SXYQ1921-3	99.6	98	99.5	99.5	99.6	99.4	***	98.7	96.8	96.6	96.7	99.7	96.8	96.9	98.5	98.6	97	97
8	SXYQ1922-2	99	97.7	98.8	98.9	98.7	98.7	98.8	***	96.6	96.6	96.6	98.8	96.7	96.7	98.2	98.5	96.7	96.8
9	SXYQ1923-4	98.4	99	98.2	98.1	98.2	98.2	98.2	97.8	***	96.5	99.2	97	99.5	99.5	97.1	96.9	96.7	96.7
10	SXYQ1941-5	98.1	97.9	97.9	97.8	97.7	97.9	97.8	97.5	97.9	***	96.5	96.7	96.6	96.6	96.6	96.6	96.9	97
11	SXYQ1944-1	98.2	98.8	97.9	97.9	97.9	97.9	97.9	97.7	98.8	97.9	***	96.8	99.2	99.3	97	96.9	96.5	96.5
12	SXYQ1964-2	99.7	98.2	99.7	99.6	99.5	99.6	99.6	98.8	98.3	98	98	***	97	97.1	98.4	98.6	97.1	97.2
13	SXYQ1965-3	98.5	99.5	98.3	98.2	98.1	98.2	98.2	97.9	99.2	98.1	99	98.4	***	99.5	97.1	97	96.9	96.8
14	SXYQ1966-2	98.5	99.3	98.4	98.2	98.2	98.3	98.2	97.9	99.3	98.2	99	98.5	99.5	***	97.1	97	96.7	96.9
15	SXYQ1966-4	99.2	97.9	99	99.1	98.8	98.8	98.9	98.8	97.9	97.7	97.8	99	98	98.1	***	99	96.8	96.8
16	SXYQ1966-5	99.4	98.4	99.1	99.3	99	99	99.1	99	98.5	98	98.3	99.2	98.5	98.6	99.4	***	96.7	96.8
17	SXYQ1968-2	97.6	97.7	97.4	97.3	97.4	97.4	97.4	97	97.5	97.6	97.2	97.5	97.9	97.7	97.1	97.5	***	99.8
18	SXYQ1968-3	97.7	97.6	97.6	97.4	97.4	97.5	97.4	97.1	97.7	97.9	97.4	97.7	97.9	97.9	97.3	97.7	99.4	***

Note: The upper right side of *** is the homology analysis of the nucleotide sequence in the coding region; The lower left side of *** is the homology comparison of amino acid sequence in the coding region

Supplement table S4. The homology analysis of nucleotide (amino acid) sequence of NS gene of WUXV viruses isolated in Yangquan County.

NO	Strain	Strain																	
		1	2	3	4	5	6	7	8	9	10	11	12	13	14	15	16	17	18
1	SXYQ1944-2	***	97.4	97.8	97.6	97.6	94.6	97.7	95.7	97.6	97.4	97.7	97.7	97.6	97.4	95.8	95.8	95.9	95.9
2	SXYQ1903	97.3	***	98.6	98.3	98.3	95.4	98.5	96.7	99.6	99.5	99.7	98.5	99.6	99.7	96.9	96.9	96.9	96.9
3	SXYQ1913-2	98.1	98.9	***	99.7	99.7	96.3	99.9	96.8	98.7	98.6	98.9	99.9	98.7	98.6	96.9	96.9	97.1	97.1
4	SXYQ1918-2	97.3	98.1	98.9	***	99.7	96	99.9	96.6	98.5	98.3	98.6	99.9	98.5	98.3	96.7	96.7	96.8	96.8
5	SXYQ1919-1	97.3	98.1	98.9	98.9	***	96	99.9	96.6	98.5	98.3	98.6	99.9	98.5	98.3	96.7	96.7	96.8	96.8
6	SXYQ1921-2	94.3	95	95.8	95	95	***	96.2	94.6	95.5	95.4	95.7	96.2	95.5	95.4	94.8	94.8	94.6	94.6
7	SXYQ1921-3	97.7	98.5	99.2	99.2	99.2	95.4	***	96.7	98.6	98.5	98.7	100	98.6	98.5	96.8	96.8	96.9	96.9
8	SXYQ1922-2	98.1	98.9	99.6	98.9	98.9	95.8	99.2	***	96.8	96.7	96.9	96.7	96.8	96.7	99.1	99.1	98.5	98.5
9	SXYQ1923-4	98.1	98.9	99.6	98.9	98.9	95.8	99.2	99.6	***	99.9	99.9	98.6	100	99.9	97.1	97.1	97.3	97.3
10	SXYQ1941-5	97.7	98.5	99.2	98.5	98.5	95.4	98.9	99.2	99.2	***	99.7	98.5	99.9	99.7	96.9	96.9	97.2	97.2
11	SXYQ1944-1	98.1	98.9	99.6	98.9	98.9	95.8	99.2	99.6	99.6	99.2	***	98.7	99.9	99.7	97.2	97.2	97.2	97.2
12	SXYQ1964-2	97.7	98.5	99.2	99.2	99.2	95.4	99.6	99.2	99.2	98.9	99.2	***	98.6	98.5	96.8	96.8	96.9	96.9
13	SXYQ1965-3	98.1	98.9	99.6	98.9	98.9	95.8	99.2	99.6	99.6	99.2	99.6	99.2	***	99.9	97.1	97.1	97.3	97.3
14	SXYQ1966-2	97.7	99.2	99.2	98.5	98.5	95.4	98.9	99.2	99.2	98.9	99.2	98.9	99.2	***	96.9	96.9	97.2	97.2
15	SXYQ1966-4	98.1	98.9	99.6	98.9	98.9	95.8	99.2	99.6	99.6	99.2	99.6	99.2	99.6	99.2	***	100	98.3	98.3
16	SXYQ1966-5	98.1	98.9	99.6	98.9	98.9	95.8	99.2	99.6	99.6	99.2	99.6	99.2	99.6	99.2	99.6	***	98.3	98.3
17	SXYQ1968-2	98.1	98.9	99.6	98.9	98.9	95.8	99.2	99.6	99.6	99.2	99.6	99.2	99.6	99.2	99.6	99.6	***	100
18	SXYQ1968-3	98.1	98.9	99.6	98.9	98.9	95.8	99.2	99.6	99.6	99.2	99.6	99.2	99.6	99.2	99.6	99.6	99.6	***

Note: The upper right side of *** is the homology analysis of the nucleotide sequence in the coding region; The lower left side of *** is the homology comparison of amino acid sequence in the coding region

Supplement table S5. The homology analysis of nucleotide (amino acid) sequence of N gene of WUXV viruses isolated in Yangquan County.

NO	Strain	Strain																	
		1	2	3	4	5	6	7	8	9	10	11	12	13	14	15	16	17	18
1	SXYQ1944-2	***	98.9	98.9	98.8	98.8	98.9	98.9	98.8	99.1	99.1	99.1	98.9	98.8	99.1	98.5	98.2	98.5	98.5
2	SXYQ1903	99.2	***	98.9	98.8	98.8	98.9	98.9	98.8	99.6	99.6	99.6	98.9	99.3	99.6	98.5	98.2	98.5	98.5
3	SXYQ1913-2	99.6	99.2	***	99.9	99.9	100	100	98.9	99.1	99.1	99.1	100	98.8	99.1	98.7	98.4	98.8	98.8
4	SXYQ1918-2	99.2	98.8	99.2	***	100	99.9	99.9	99.1	98.9	98.9	98.9	99.9	98.7	98.9	98.5	98.5	98.7	98.9
5	SXYQ1919-1	99.2	98.8	99.2	99.6	***	99.9	99.9	99.1	98.9	98.9	98.9	99.9	98.7	98.9	98.5	98.5	98.7	98.9
6	SXYQ1921-2	99.6	99.2	99.6	99.2	99.2	***	100	98.9	99.1	99.1	99.1	100	98.8	99.1	98.7	98.4	98.8	98.8
7	SXYQ1921-3	99.6	99.2	99.6	99.2	99.2	99.6	***	98.9	99.1	99.1	99.1	100	98.8	99.1	98.7	98.4	98.8	98.8
8	SXYQ1922-2	99.2	98.8	99.2	99.6	99.6	99.2	99.2	***	98.9	98.9	98.9	98.9	98.7	98.9	98.9	99.2	99.1	99.1
9	SXYQ1923-4	99.6	99.2	99.6	99.2	99.2	99.6	99.6	99.2	***	99.7	99.7	99.1	99.7	99.7	98.7	98.4	98.7	98.7
10	SXYQ1941-5	99.6	99.2	99.6	99.2	99.2	99.6	99.6	99.2	99.6	***	99.7	99.1	99.5	100	98.7	98.4	98.7	98.7
11	SXYQ1944-1	99.6	99.2	99.6	99.2	99.2	99.6	99.6	99.2	99.6	99.6	***	99.1	99.5	99.7	98.7	98.4	98.7	98.7
12	SXYQ1964-2	99.6	99.2	99.6	99.2	99.2	99.6	99.6	99.2	99.6	99.6	99.6	***	98.8	99.1	98.7	98.4	98.8	98.8
13	SXYQ1965-3	99.6	99.2	99.6	99.2	99.2	99.6	99.6	99.2	99.6	99.6	99.6	99.6	***	99.5	98.4	98.1	98.4	98.4
14	SXYQ1966-2	99.6	99.2	99.6	99.2	99.2	99.6	99.6	99.2	99.6	99.6	99.6	99.6	99.6	***	98.7	98.4	98.7	98.7
15	SXYQ1966-4	99.6	99.2	99.6	99.2	99.2	99.6	99.6	99.2	99.6	99.6	99.6	99.6	99.6	99.6	***	99.7	98.5	98.5
16	SXYQ1966-5	99.2	98.8	99.2	99.6	99.6	99.2	99.2	99.6	99.2	99.2	99.2	99.2	99.2	99.2	99.2	***	98.5	98.5
17	SXYQ1968-2	99.6	99.2	99.6	99.2	99.2	99.6	99.6	99.2	99.6	99.6	99.6	99.6	99.6	99.6	99.6	99.2	***	99.7
18	SXYQ1968-3	99.2	98.8	99.2	99.6	99.6	99.2	99.2	99.6	99.2	99.2	99.2	99.2	99.2	99.2	99.2	99.6	99.2	***

Note: The upper right side of *** is the homology analysis of the nucleotide sequence in the coding region; The lower left side of *** is the homology comparison of amino acid sequence in the coding region
